# SLC7A11 as a therapeutic target to attenuate phthalates-driven testosterone level decline in mice

**DOI:** 10.1016/j.jare.2024.05.026

**Published:** 2024-05-24

**Authors:** Yi Zhao, Xue-Qi Wang, Rui-Qi Liu, Fu-Wei Jiang, Jia-Xin Wang, Ming-Shan Chen, Hao Zhang, Jia-Gen Cui, Yuan-Hang Chang, Jin-Long Li

**Affiliations:** a College of Veterinary Medicine, Northeast Agricultural University, Harbin 150030, PR China; b Key Laboratory of the Provincial Education Department of Heilongjiang for Common Animal Disease Prevention and Treatment, Northeast Agricultural University, Harbin 150030, PR China; c Heilongjiang Key Laboratory for Laboratory Animals and Comparative Medicine, Northeast Agricultural University, Harbin 150030, PR China

**Keywords:** Di (2-ethylhexyl) phthalate, Solute carrier family 7 member 11, Ferroptosis, Testosterone, Male mice

## Abstract

**Introduction:**

Phthalates exposure is a major public health concern due to the accumulation in the environment and associated with levels of testosterone reduction, leading to adverse pregnancy outcomes. However, the relationship between phthalate-induced testosterone level decline and ferroptosis remains poorly defined.

**Objectives:**

Herein, we aimed to explore the mechanisms of phthalates-induced testosterone synthesis disorder and its relationship to ferroptosis.

**Methods:**

We conducted validated experiments in vivo male mice model and in vitro mouse Leydig TM3 cell line, followed by RNA sequencing and metabolomic analysis. We evaluated the levels of testosterone synthesis-associated enzymes and ferroptosis-related indicators by using qRT-PCR and Western blotting. Then, we analyzed the lipid peroxidation, ROS, Fe^2+^ levels and glutathione system to confirm the occurrence of ferroptosis.

**Results:**

In the present study, we used di (2-ethylhexyl) phthalate (DEHP) to identify ferroptosis as the critical contributor to phthalate-induced testosterone level decline. It was demonstrated that DEHP caused glutathione metabolism and steroid synthesis disorders in Leydig cells. As the primary metabolite of DEHP, mono-2-ethylhexyl phthalate (MEHP) triggered testosterone synthesis disorder accompanied by a decrease in the expression of solute carri1er family 7 member 11 (SLC7A11) protein. Furthermore, MEHP synergistically induced ferroptosis with Erastin through the increase of intracellular and mitochondrial ROS, and lipid peroxidation production. Mechanistically, overexpression of SLC7A11 counteracts the synergistic effect of co-exposure to MEHP-Erastin.

**Conclusion:**

Our research results suggest that MEHP does not induce ferroptosis but synergizes Erastin-induced ferroptosis. These findings provide evidence for the role of ferroptosis in phthalates-induced testosterone synthesis disorder and point to SLC7A11 as a potential target for male reproductive diseases. This study established a correlation between ferroptosis and phthalates cytotoxicity, providing a novel view point for mitigating the issue of male reproductive disease and “The Global Plastic Toxicity Debt”.

## Highlights

• DEHP induces testosterone synthesis disorder in the testis.
• MEHP impairs mitochondrial structure and function in Leydig cells.
• MEHP disrupts glutathione metabolism in Leydig cells.
• MEHP aggravates ferroptosis by downregulating SLC7A11 expression.
• SLC7A11 acts as an effector in blocking MEHP-Erastin-induced ferroptosis.

## Graphical abstract

DEHP causes structural and functional damage to Leydig cell, and synergistically induces ferroptosis by decreasing SLC7A11 level in Leydig cells. Regulation SLC7A11 levels exhibits potential in treating ferroptosis-induced male reproductive disease.

**Figure:**
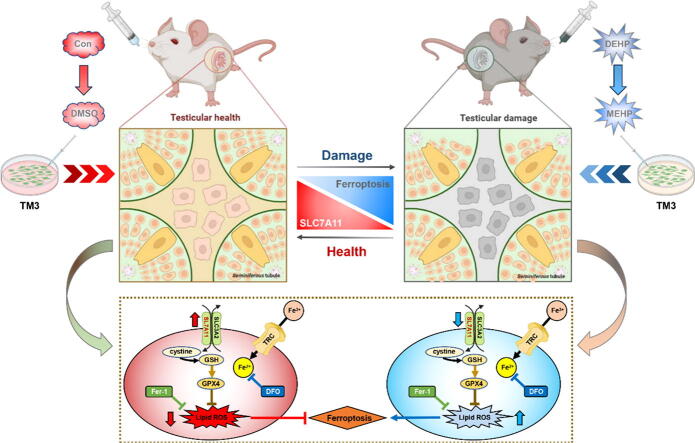

## Introduction

Phthalates presence in agriculture and industry has been widely been recognized as a potential threat to human health, and are associated with etiopathogenesis that adversely effects on male reproductive function [1]. As the most abundantly used and ubiquitous phthalates, di (2-ethylhexyl) phthalate (DEHP) is mainly used as plasticizer in polyvinyl chloride products to improve the strength and plasticity of plastic products, especially plastic film and other agricultural plastic products [2]. Although the production of DEHP is decreasing owing to negative impacts on health, it is predicted to account for nearly one third of 10 million tons of the global plasticizer market in 2024 [3]. A recent study indicated that toxicity of DEHP exposure to wheat seedlings was dose-dependent because of its ecotoxicological impacts on organisms [4]. Accordingly, DEHP accumulates in the body through the food chain, and disrupting organ function [5]. Some previous studies reported the correlation between DEHP exposure and male infertility [6]. DEHP is associated with ‘phthalate syndrome’ of male reproductive system as recognized by the National Academy of Science [7]. The male reproductive adverse effects of DEHP have garnered significant attention recently owing to growing public awareness of environmental issues.

The hypothalamic-pituitary-testis (HPT) axis regulates the normal male reproductive functions and sexsteroid productions. Testosterone (T) as a reproductive hormone is produced during steroid synthesis and promotes spermatogenesis [8]. A large number of studies discovered that environmental contaminants including polychlorinated biphenyls (PCBs) and phthalate have proven to be negatively related to testosterone level [9], [10]. Leydig cells are steroidogenic cells present in the interstitial compartment of the testes and has ability to produce testosterone synthesis-related enzymes and molecules. It has been suggested that the environmental contaminants could cause damage to Leydig cell structure, reduced Leydig cell steroidogenesis, and negatively affected testicular function [11]. However, there are still needed systematic researches to prove the effects of DEHP on testosterone synthesis and its underlying mechanisms remains poorly understood.

Ferroptosis is novel form of cellular death caused by impairment of selective permeability of plasma membrane as a result of pronounced peroxidation of membrane lipids [12]. System Xc^-^ is an antiporter consists of two vital components, including solute carrier family 7 member 11 (SLC7A11) and SLC3A2 [13]. The activity of system Xc^-^ is involved in the maintenance of intracellular glutathione levels, which protect cells against ferroptosis [14]. SLC7A11 is a multichannel transmembrane protein that regulates cystine/glutamate antiporter activity in the system Xc^-^ [15]. This antiporter is used to synthesize the main antioxidant glutathione (GSH) by reducing cystine to cysteine, thereby maintaining the intracellular antioxidant defense system. In addition, certain small molecule compounds (Erastin) can induce ferroptosis by inhibiting systemic Xc- system, which is a non-apoptotic kind of lipid peroxide-dependent regulation of cell death [16]. Numerous studies have identified that regulating SLC7A11 expression is favorable for disease therapy, and thus SLC7A11 is regarded as a potential therapeutic target. Furthermore, SLC7A11 represents a better therapeutic target for disease treatment than GPX4, because inhibiting SLC7A11 could presumably cause less toxicity inpatients than inhibiting GPX4 [17]. However, the potential regulatory role of SLC7A11 in testicular injury and its association with ferroptosis are poorly understood.

Although there is novel evidence suggested that DEHP may be involved in cell death such as autophagy [18], apoptosis [19] and pyroptosis [20], but precise role and mechanism remains unclear whether and how DEHP is involved in ferroptosis. Several studies have confirmed that a negative correlation was established between DEHP levels and sperm quality, along with positive associations with depolarized mitochondria, elevated ROS production, and lipid peroxidation [21], [22]. Therefore, the present study aims to explore the potential molecular mechanisms of DEHP-induced testosterone synthesis disorder and its association with ferroptosis, and determine the potential role of SLC7A11 in it. In this study, we have proved that DEHP induces damage to Leydig cells by disrupting the HPT and inhibiting testosterone synthesis. Interestingly, mono(2-ethylhexyl) phthalate (MEHP) synergistically induce ferroptosis with Erastin, such as increased lipid peroxidation and ferrous ion levels, and reduced GSH. We also analyzed ferroptosis-related markers in Eratin-induced ferroptosis model after exposure to MEHP, and only SLC7A11 and GPX4 were significantly inhibited. To investigate the molecular mechanism of MEHP in ferroptosis, we focused on SLC7A11. Consistent with the results obtained with ferroptosis inhibitors, SLC7A11 overexpression could rescue combined exposure-induced ferroptosis via maintaining GSH in Leydig cells, further demonstrates the crucial role of SLC7A11-dependent pathway in ferroptosis process. The discovery of SLC7A11 as a pivotal modular of ferroptosis provides opportunities to improve ferroptosis-based male reproductive disease therapy.

## Materials and methods

### Ethics statement

All procedures for animal experiments were performed in the Guidelines for Care and Use of Laboratory Animals of Northeast Agricultural University (NEAU). Research experiments were approved by the Animal Ethics Committee (NEAUEC20220341). All animal experiments comply with the ARRIVE guidelines and International Guiding Principles for Biomedical Research Involving Animals.

### In vivo mouse studies

The male ICR mice, 3-weeks-old, were acquired from Liaoning Changsheng Biotech Co., Ltd. Mice were housed in standard cages at 22 ± 2 °C (50 ± 15 % humidity) with a 12/12 h day/night cycle, and were provided basal diet and water ad libitum. Mice were randomly divided into 5 groups and details are shown in Table S1. Mice were treated by DEHP (Aladdin, Shanghai, China) for seven weeks by oral gavage. The lowest observed adverse effect level of DEHP was 140 mg/kg [23]. The LD_50_ of DEHP in rodents is 30 g/kg and the dose of DEHP in this study was similar to the previous research, at the doses of 50 mg/kg (1/600 LD50), 200 mg/kg (1/150 LD50), and 500 mg/kg (1/60 LD50) [24], [25], [26]. DEHP (200 mg/kg/d) causes DNA damage in sperm and has detrimental effects on the reproductive system from the prepubertal to the pubertal period [27]. DEHP (500 mg/kg/d) induces spermatogenesis disorder by inhibiting testosterone/androgen receptor pathway [6]. The DEHP in soil (1–264 mg/kg) or sewage sludge (12–1250 mg/kg) is difficult to degrade and thus persist in the environment [28]. Furthermore, daily intake of DEHP for critically ill preterm infants reaches 16 mg/kg per day, which is equivalent to 200 mg/kg in mice [29].

### Cell culture

TM3 cell was purchased from the Cell Bank of Type Culture Collection of the Chinese Academy of Sciences (Shanghai, China), and were cultured in DMEM/F12 (HyClone, USA; Meilunbio, China) supplemented with 10 % FBS (VivaCell, Shanghai, China) and 1 % penicillin–streptomycin (Thermos, USA; Yeasen, Shanghai, China). Culture flasks and plates were purchased from NEST Biotechnology Co., Ltd. 10 μΜ Erastin, 1 μΜ Fer-1, 0.5 μΜ DFO (MedChemExpress, USA), and MEHP (AccuStandard, USA) were dissolved in DMSO and subsequently diluted with DMEM/F12. The final concentration of DMSO did not exceed 0.1 %, thus no cytotoxicity was observed in this concentration.

### Rna-seq analysis

RNA-seq was performed according to the previous method [30]. RNA-seq and bioinformatics analyses were executed by ShangHaiWeiHuan BiotechCo., Ltd and Shenggong Bioengineering Co., Ltd (Shanghai, China). The detailed methods were shown in Supplementary Methods 1.1.

### Metabolite profiling analysis

LC/MS detection was performed in metabolite profiling analysis following the previous methods [31]. Metabolite profiling analysis was executed by ShangHaiWeiHuan BiotechCo., Ltd (Shanghai, China). The detailed methods were shown in Supplementary Methods 1.2.

### Cell transfection

The expression vector (SLC7A11 overexpression) was obtained from Shenggong Bioengineering Co., Ltd (Shanghai, China). In brief, when the cell confluency was 50 %-70 %, TM3 cells were transfected with 2.5 μg of pcDNA3.1-SLC7A11 vector, 5 μL of P3000, and 5 μL of Lipofectamine 3000, and the whole pcDNA3.1 plasmid was transfected as a negative control. Subsequently, the cells were incubated for 24 h.

### Transmission electron microscopy (TEM)

The tissues and cells were fixed with glutaraldehyde solution. The samples were treated with osmic acid treatment, acetone dehydration, resin embedding, and lead citrate staining. The sections were imaged with an electron microscopy (TEM, H-7650, Tokyo, Japan). Flameng score and mitochondrial average surface area were measured by using Image J software (https://imagej.net/ij/docs/faqs.html#cite) [32], [33]. The mitochondrial Flameng score criteria under TEM was shown in detail in Supplementary Methods 1.3.

### Hormone related index

To evaluate the hormone levels in DEHP-induced mice, serum T, P, E_2_, PRL, LH and, FSH levels and cellular supernatant T levels were measured by ELISA kit (Meimian Industrial, Jiangsu, China) according to the manufacturer's protocol.

### Cell viability assay

Cell viability of TM3 cells was evaluated using a CCK8 (Dojindo, Tokyo, Japan). In brief, 5000 cells were spread in a 96-well cell culture plate. Then, CCK8 reagent was added and absorbance was detected at 450 nm.

### qPCR analysis

The total RNA was isolated from testicular tissues and TM3 cells as described in previous methods and detail in Supplementary Methods 1.4 [34], [35]. The mRNA levels of GADPH and β-actin were used for normalization. Primer sequences used are listed in Table S2.

### Western blot analysis

Western blot analysis was performed as previously reported [36], [37]. The total protein was extracted from tissues using RIPA lysis Buffer (APExBIO, Houston, USA; BioChannel, China) supplemented with PMSF (Biochannel, Nanjing, China; Seven, Beijing, China) and protease inhibitor cocktail (MedChem Express, USA) according to the previous study [38]. Primary antibodies were provided by Abcam Co., Ltd. (Cambridge, England), GeneTex Co., Ltd. (Irvine, CA, USA), ABclonal Co., Ltd. (Wuhan, China), Bioss-Bio Co., Ltd. (Beijing, China), Proteintech Co., Ltd. (Chicago, USA) (Table S3). Secondary antibodies were obtained from ZSGB-Bio Co., Ltd. (Beijing, China). The detailed methods were shown in Supplementary 1.5.

### Glutathione system measurement

The levels of GSH/GSSG, T-GSH, GSH-PX, and GSH-ST were measured from protein of TM3 cells using commercial kits (Jiancheng Biotech, Nanjing, China).

### Lipid peroxidation and DNA oxidative damage assay

The levels of MDA, LPO and 8-OH were measured from protein of TM3 cells using commercial kits (Jiancheng Biotech, Nanjing, China).

### Measurement of intracellular ROS

The level of intracellular ROS was detected with a DCFH-DA probe (Solaibao, Beijing, China). In brief, the DCFH-DA was added to 12-well plate, which was incubated for 30 min. The results were analyzed using the fluorescence microscope (Leica, Germany).

### Measurement of lipid ROS

To analyze lipid ROS, TM3 cells were stained with C11-BODIPY581/591 (Thermo, USA) for 30 min. The lipid ROS was observed under a fluorescence microscope.

### Measurement of mitochondrial ROS (mtROS)

TM3 cells were incubated with MitoTracker Green (Beyotime, China) at 37 °C for 30 min and MitoSox Red (Thermo, USA) for 10 min. TM3 cells were observed by using a fluorescence microscope.

### Cytoplasmic and mitochondrial Fe^2+^ level measurement

The level of Fe^2+^ was detected using FerroOrange and MitoFerroGreen (Dojindo Molecular Technologies, Japan). The FerroOrange and MitoFerroGreen were added to the TM3 cells and incubated for 0.5 h. The TM3 cells were observed using a fluorescence microscope.

### Construction of PPI network

The STRING (Search Tool for the Retrieval of Interacting Proteins) database (https://string-db.org) was used for PPI networks.

### Molecular docking

The molecular docking was performed by AutoDock software, and the visualization was performed by PyMOL software. Molecular docking was described in detail in Supplementary Methods 1.6.

### Statistical analysis

The data from at least three experiments were presented as means ± SD. One-way analysis of variance (ANOVA) was used to perform the comparisons and followed by Tukey's multiple comparison tests on dependent experimental designs. *P* < 0.05 was considered statistically significant.

## Results

### DEHP drove the testosterone level decline in Leydig cells

**Fig. 1:**
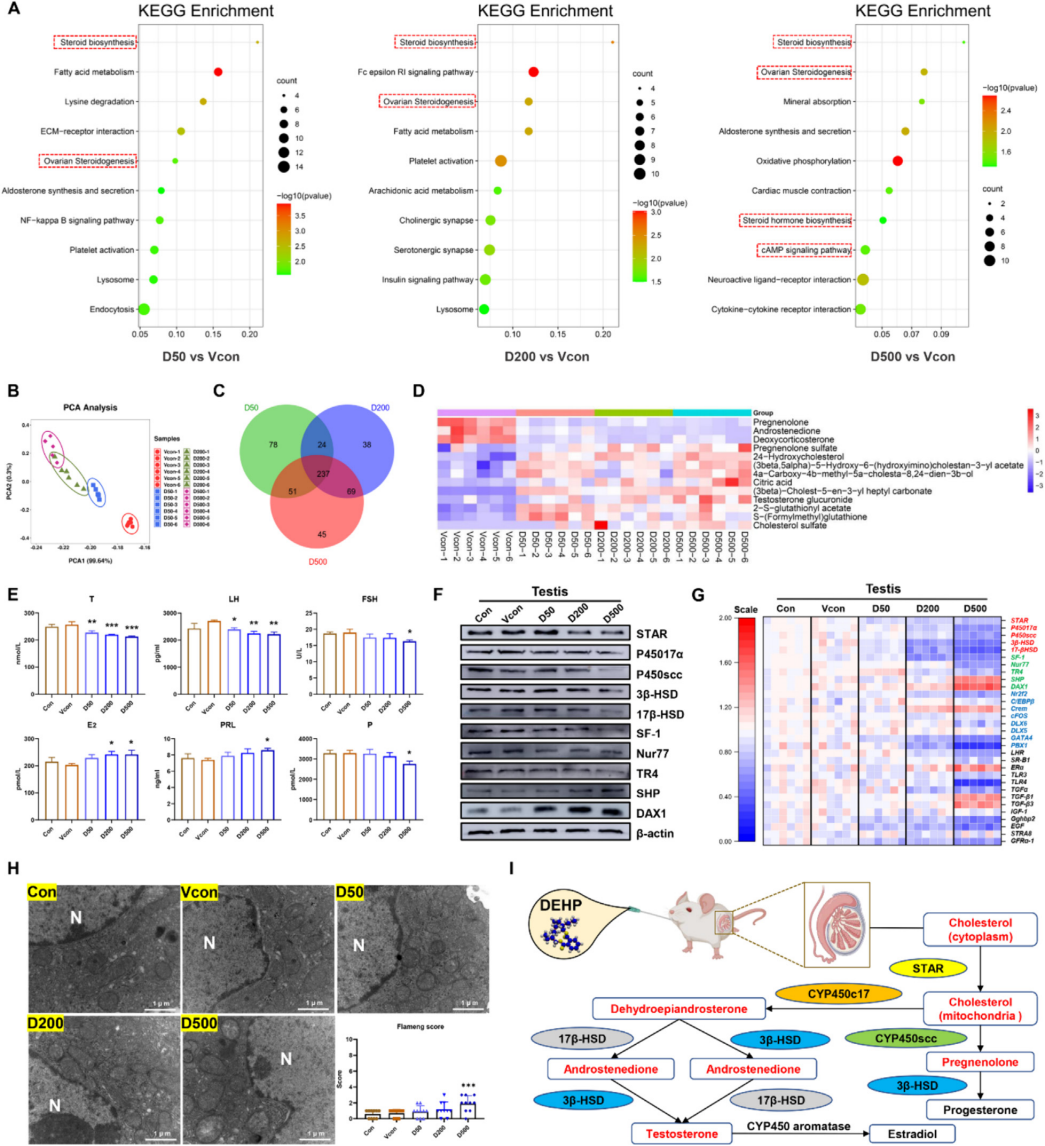
**DEHP induced HPT axis disorder in the mouse Leydig cells (n = 3).** (A) KEGG analysis. (B) PCA analysis. (C) Venn diagram analysis. (D) Heatmap presentation of differential metabolites in steroid hormone biosynthesis. (E) The T, LH, FSH, E_2,_ PRL and P level of gonadal hormones in the mice. (F) The protein levels of testosterone synthases and hormones nuclear receptors. (G) Heatmap presentation of the mRNA levels of testosterone synthases and hormones nuclear receptors. (H) Transmission electron microscopy and Flameng score of the mice testis. (I) Schematic depicting the regulation of DEHP induced dysfunction of steroid hormone bio-anabolism in the testis of mice. Data are presented as the mean ± SD. Symbol for the significance of differences between the Vcon group and another group: *P < 0.05, **P < 0.01, ***P < 0.001.

Numerous previous studies demonstrated that phthalate exposure could affect human male reproductive health, however the precise mechanisms are yet to be elucidated further. In transcriptional profiling, KEGG analysis also suggested that DEGs were all enriched in the steroid biosynthesis and ovarian steroidogenesis pathway in DEHP groups (Fig. 1A). Testosterone is an anabolic steroid that is produced primarily in Leydig cell of the testes. Based on KEGG analyses, we hypothesized that DEHP exposure might cause testosterone synthesis disorder and testosterone level decline. Meanwhile, nontargeted metabolic profiling and Venn diagram analysis also indicated that steroid hormone biosynthesis significantly changed, and associated metabolites showed obvious alteration in DEHP groups (Fig. 1B-D). The main metabolite in the pathway of steroid hormone biosynthesis were sex hormones [39]. Interfering with the HPT axis may be considered as a potential mechanism for a decrease in serum T levels [40]. Consistently, the results suggested that DEHP exhibited an obvious a reduction in testosterone, P, PRL and FSH levels, while elevated the levels of E_2_ and LH in serum (Fig. 1E). Inhibiting the level or activity of steroid-producing enzymes is another underlying mechanism for reducing testosterone level [41]. Testosterone biosynthesis is a complex process in which the key enzymes STAR, P450c17, P450scc, 3β-HSD and 17β-HSD are involved [42]. Our data suggested that protein translation occurred tardily, as shown by the obvious decrease in STAR, P450c17, P450scc, 3β-HSD and 17β-HSD levels after DEHP exposure (Fig. 1F and Fig. S2C). Orphan nuclear receptors, such as SF-1, Nur77, TR4, SHP and DAX1, serve as an essential regulator of multiple hormone-induced genes in the endocrine system [43]. The results showed a significant decrease in SF-1, Nur77, TR4 and the sharp increase in SHP and DAX1 levels after DEHP exposure (Fig. 1F and Fig. S2C). Moreover, the mRNA expression occurred an obvious change in testosterone secretion-related genes level after DEHP exposure (Fig. 1G).

Testosterone is synthesized from cholesterol in Leydig cell and maintains its function [44]. Therefore, we evaluated the ultrastructure of Leydig cells following DEHP treatment. Fig. 1H depicted the ultrastructural results of Leydig cell in various groups. The decreased mitochondrial cristae and incomplete mitochondrial membrane were observed in DEHP treatment groups. Extent of mitochondrial damage was quantified using the Flameng score. We found that the Flameng score was upregulated after DEHP treatment, suggesting DEHP could induce mitochondrial damage in Leydig cells. Consistently, GO enrichment and GSEA analysis were point to male reproductive process and mitochondrial structure and function (Fig. S2A-B). Therefore, we hypothesized that DEHP exposure might affect testosterone synthesis and testosterone level decline because of mitochondrial dysfunction in Leydig cells.

**Fig. 2:**
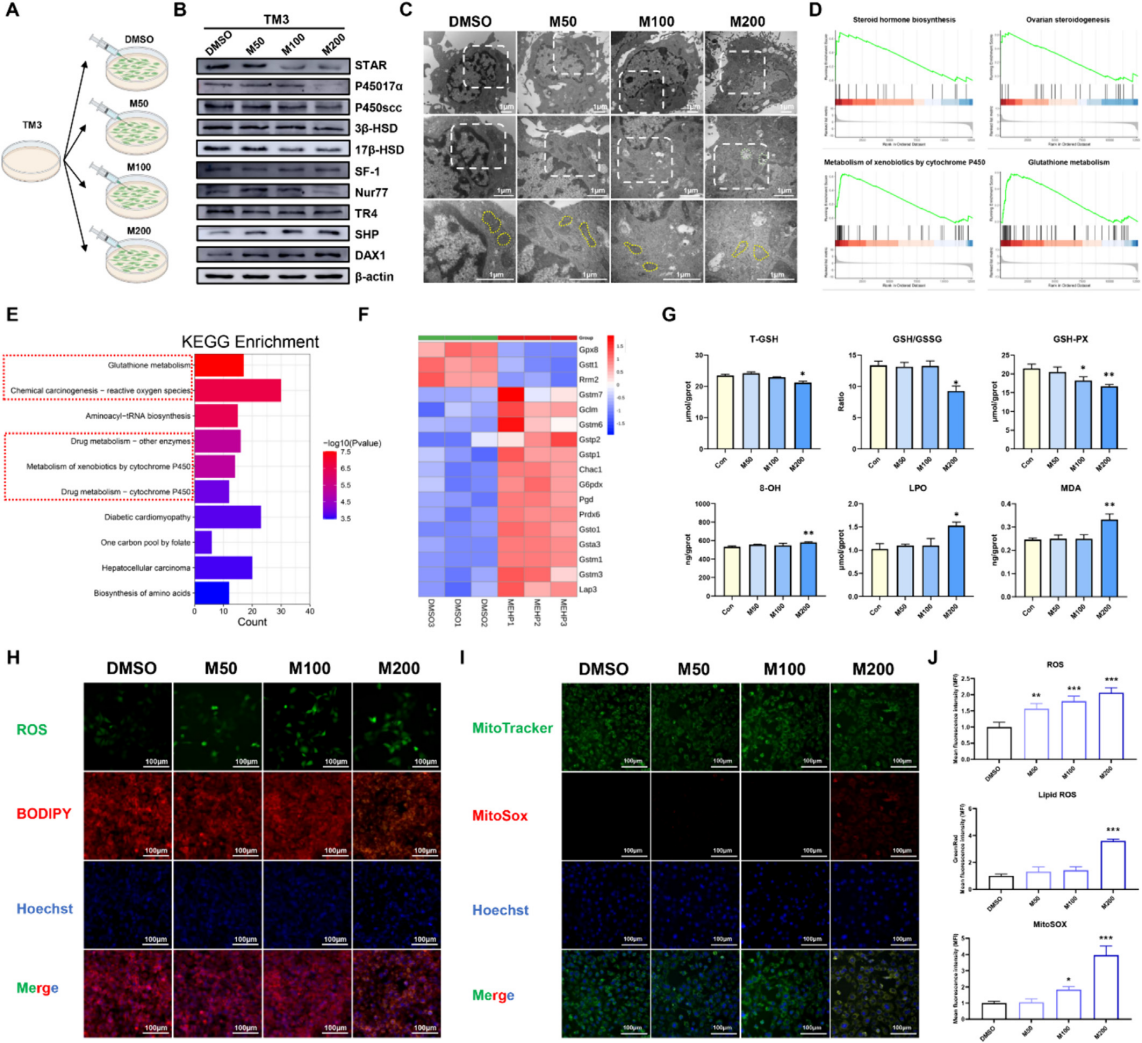
**MEHP induced disorder on the HPT axis in the Leydig cells (n = 3).** (A) TM3 cells were incubated with different concentrations of MEHP. (B) The protein levels of testosterone synthases and hormones nuclear receptors in the TM3 cells. (C) Transmission electron microscopy and Flameng score of the TM3 cells. (D) GSEA analysis based on RNA-seq of TM3 cells after exposure to M200. (E) KEGG analysis based on RNA-seq of TM3 cells after exposure to M200. (F) Heatmap shows differentially expressed genes in the glutathione metabolism. (G) The T-GSH, GSH/GSSG, GSH-PX, 8-OH, LPO, MDA levels of TM3 cells. (H) The intracellular and mitochondrial ROS of TM3 cells. (I) The lipid ROS production of TM3 cells. (J) The mean fluorescence intensity (MFI) of ROS, Lipid ROS and MitoSOX in TM3 cells. Data are presented as the mean ± SD. Symbol for the significance of differences between the DMSO group and another group: *P < 0.05, **P < 0.01, ***P < 0.001.

DEHP is in vivo metabolized quantitatively to its monoester mono-2-ethylhexyl phthalate (MEHP), which is the vital metabolite of DEHP and considered to be more toxic than DEHP [45]. To further evaluate the impact of DEHP on the Leydig cell, we measured markers of testosterone synthesis following MEHP treatment in TM3 cell line. We chose 50, 100 and 200 μM MEHP for subsequent experiments (Fig. 2A and Fig. S3A). A few mitochondrial cristae were not clear and a few numbers of autophagic vesicles were observed in MEHP groups (Fig. 2C). Nevertheless, there was no significant difference in Flameng score between the groups treated with DMSO and MEHP (Fig. S3B). Consistent with the vivo results, our data indicated that MEHP changes the testosterone-related enzymes and transcription factors level in TM3 cells (Fig. 2B, Fig. S3C-D). The result proved that DEHP exposure resulted in the testosterone level decline by orphan nuclear receptors and testosterone enzymes dysregulation (Fig. 1I).

### MEHP induced glutathione metabolism disorder in TM3 cells

In the vitro study, we chose MEHP (200 μM) for transcriptomic analysis (Fig. S1). Consistent with the vivo results, GSEA analyses showed four significantly enriched pathways including steroid hormone biosynthesis, ovarian steroidogenesis, metabolism of xenobiotics by cytochrome P450 and glutathione metabolism in M200 group (Fig. 2D). Of note, the KEGG enrichment, GO analyses and GSEA analyses results suggested that DEGs were mainly enriched in the glutathione metabolism after exposure to MEHP (Fig. 2D-F and Fig. S3E). Therefore, it is speculated that MEHP-induced testosterone level decline is associated with glutathione metabolism disorder in TM3 cells. The function of glutathione is a protective mechanism against ROS [46]. Subsequently, we investigate whether MEHP leads to glutathione system disorder and lipid peroxides accumulation. As expected, MEHP exposure significantly reduced GSH-PX, T-GSH and GSH/GSSG levels, and enhanced 8-OH, LPO and MDA generation (Fig. 2G). Moreover, the level of 4-HNE was obviously enhanced following MEHP treatment (Fig. S4D-E). The findings also indicated that MEHP could increase intracellular, lipid and mitochondrial ROS production in TM3 cells (Fig. 2G-J).

Ferroptosis is triggered by the inactivation of GSH system and accumulation of lipid peroxides [47]. Our results demonstrated that MEHP induced lipid peroxidation and GSH system disorder in TM3 cells, which probably represent the activation of ferroptosis. To identify whether ferroptosis was activated in the TM3 cells, we measured the related markers of ferroptosis upon MEHP treatment. The data readily demonstrated that the levels of SLC7A11was significantly reduced after MEHP exposure as depicted (Fig. S4D-E). We observed no obvious difference in levels of other ferroptosis-related markers between DMSO and MEHP treatment group (Fig. 2H-J, Fig. S3F, Fig. S4A and D-E). Moreover, MEHP did not affect iron levels in both cytoplasm and mitochondria, which identified by FerroOrange and MitoFerroGreen probes (Fig. S4B-C and F-G). As a whole, these findings suggested that MEHP can induce GSH system disorder and lipid peroxidation accumulation in TM3 cells, but MEHP does not cause iron-dependent ferroptosis.

### MEHP synergistically induced ferroptosis with Erastin by inhibiting SLC7A11

**Fig. 3:**
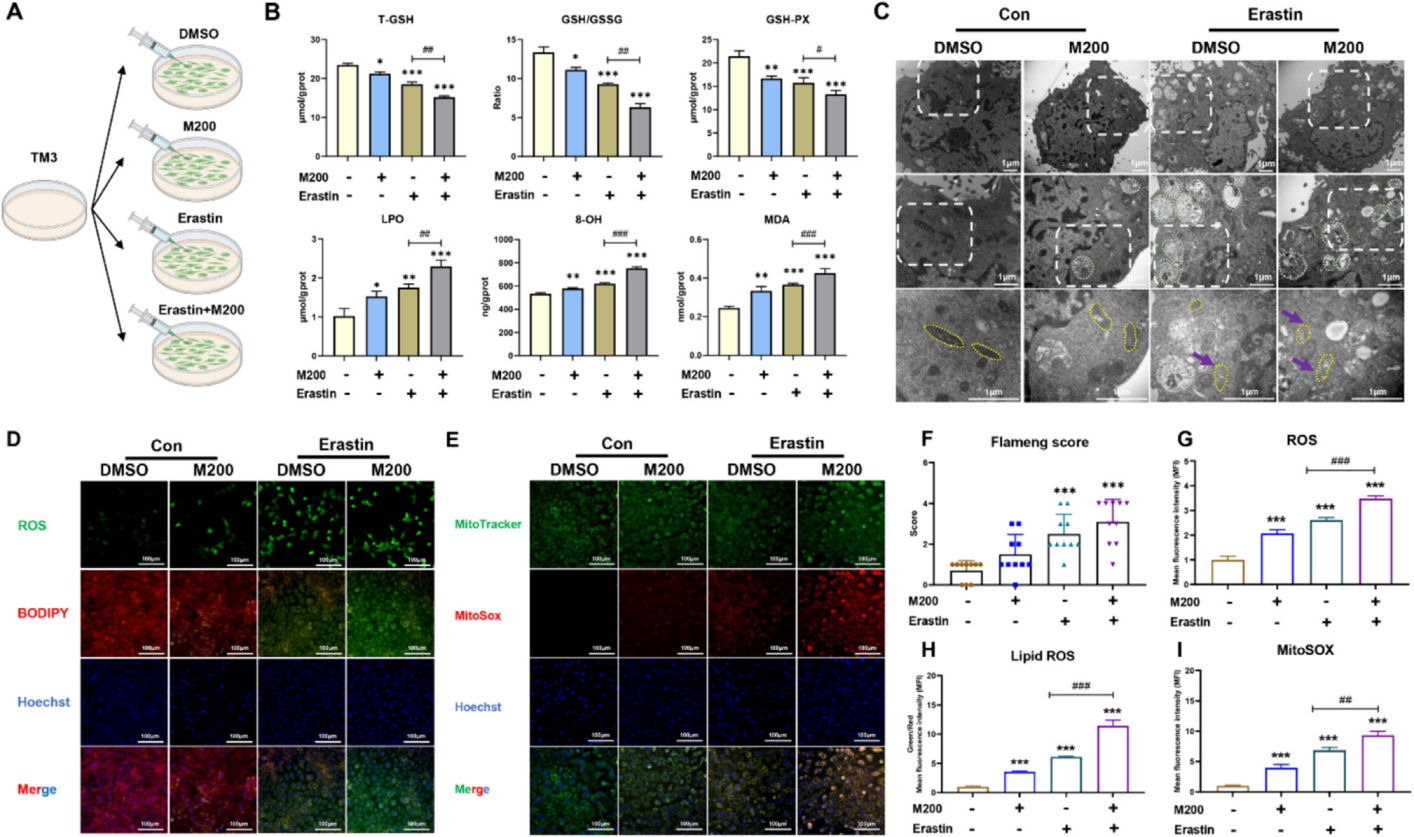
**MEHP synergistically induced ferroptosis with Erastin through lipid peroxidation in TM3 cells (n = 3).** (A) TM3 cells were incubated with DMSO, M200, Erastin or Erastin-MEHP. (B) The T-GSH, GSH/GSSG, GSH-PX, 8-OH, LPO, MDA levels of TM3 cells treated with DMSO, M200, Erastin or Erastin-MEHP. (C) Transmission electron microscopy of the TM3 cells; green: autophagic vacuole; yellow: mitochondria; purple arrow: damaged mitochondria. (D) The intracellular and lipid ROS production in TM3 cells. (E) The mitochondrial ROS production in TM3 cells. (F) The flameng score of TM3 cells (G) The mean fluorescence intensity (MFI) of ROS in TM3 cells. (H) The mean fluorescence intensity (MFI) of Lipid ROS in TM3 cells. (I) The mean fluorescence intensity (MFI) of MitoSOX in TM3 cells. Data are presented as the mean ± SD. Symbol for the significance of differences between the DMSO group and other group: *P < 0.05, **P < 0.01, ***P < 0.001. Symbol for the significance of differences between the Erastin group and MEHP + Erastin (E + M) group: ^##^*P* < 0.01, ^###^*P* < 0.001. (For interpretation of the references to colour in this figure legend, the reader is referred to the web version of this article.)

**Fig. 4:**
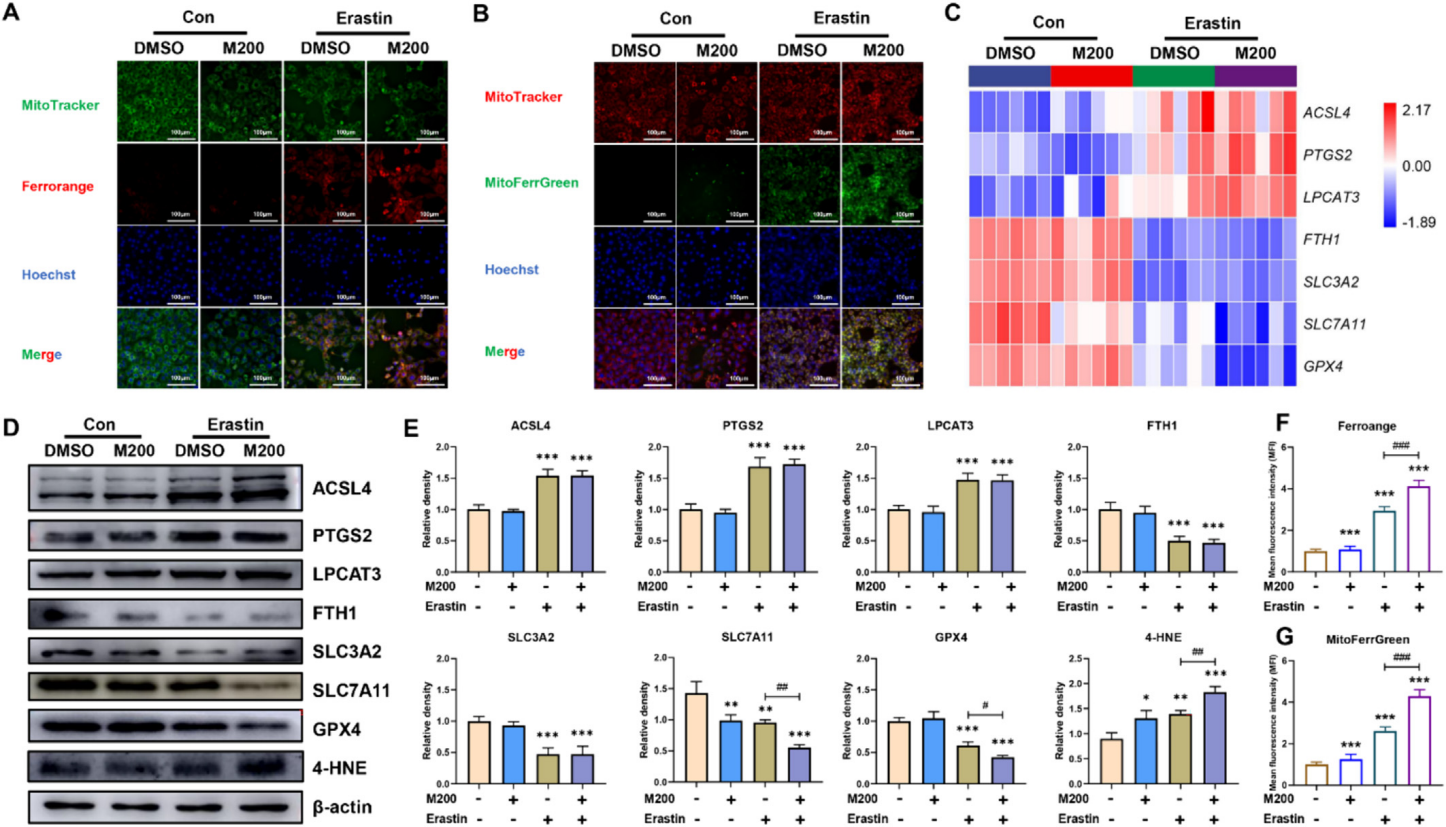
**MEHP synergistically induced ferroptosis with Erastin by increasing ferroptosis-related proteins and iron levels in TM3 cells (n = 3).** (A) The intracellular iron level of TM3 cells. (B) The mitochondrial iron level of TM3 cells. (C) Heat map of relative mRNA levels of ferroptosis related genes in the TM3 cells. (D) The protein levels of ferroptosis in the TM3 cells; β-actin is served as loading controls for the total fraction. (E) Relative protein levels of ferroptosis in the TM3 cells. (F) The mean fluorescence intensity (MFI) of Ferrorange in TM3 cells. (G) The mean fluorescence intensity (MFI) of MitoFerrGreen in TM3 cells. Data are presented as the mean ± SD. Symbol for the significance of differences between the DMSO group and other group: *P < 0.05, **P < 0.01, ***P < 0.001. Symbol for the significance of differences between the Erastin group and MEHP + Erastin (E + M) group: ^##^*P* < 0.01, ^###^*P* < 0.001.

**Fig. 5:**
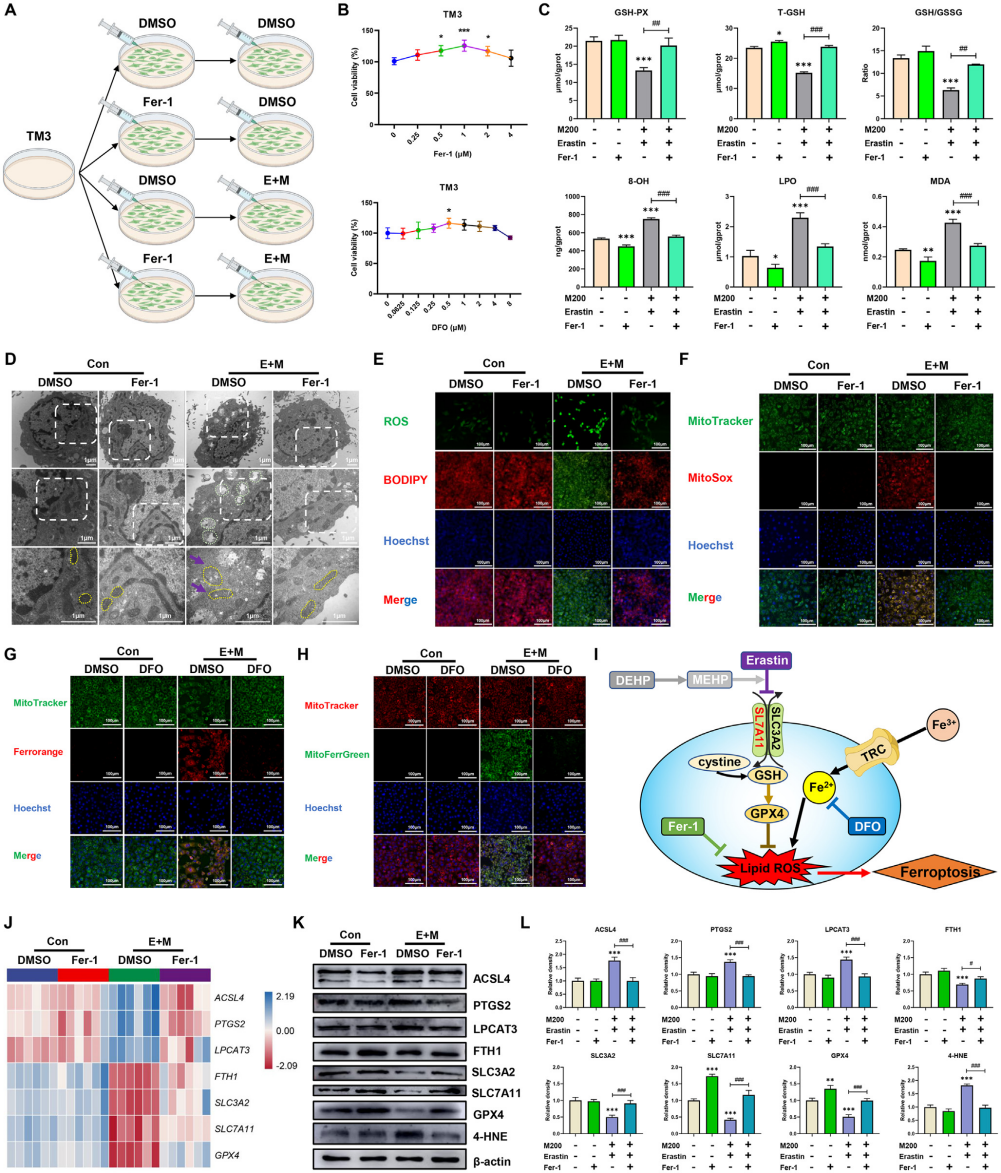
**Fer-1 and DFO inhibited MEHP-Erastin-induced ferroptosis with lipid peroxidation in TM3 cells (n = 3).** (A) TM3 cells were incubated with DMSO, Fer-1/DFO and/or Erastin-MEHP. (B) The cell viability of TM3 cells. (C) The T-GSH, GSH/GSSG, GSH-PX, 8-OH, LPO, MDA levels of TM3 cells. (D) TEM and Flameng score of the TM3 cells; green: autophagic vacuole; yellow: mitochondria; purple arrow: damaged mitochondria. (E) The intracellular and lipid ROS production of TM3 cells. (F) The mitochondrial ROS production of TM3 cells. (G) The intracellular iron level in TM3 cells. (H) The mitochondrial iron level in TM3 cells. (I) Schematic depicting the regulation of MEHP aggravating Erastin-induced ferroptosis in TM3 cells. (J) Heat map of relative mRNA levels of ferroptosis related genes in TM3 cells. (K) The protein levels of ferroptosis in the TM3 cells; β-actin is served as loading controls for the total fraction. (L) Relative protein levels of ferroptosis in the TM3 cells. Data are presented as mean ± SD. Symbol for the significance of differences between the DMSO group and another group: *P < 0.05, **P < 0.01, ***P < 0.001. Symbol for the significance of differences between the MEHP + Erastin (E + M) groups and Fer-1 + MEHP + Erastin (F + E + M) g group: ^#^*P* < 0.05, ^##^*P* < 0.01, ^###^*P* < 0.001. (For interpretation of the references to colour in this figure legend, the reader is referred to the web version of this article.)

Certain small molecular compounds (Erastin) executed ferroptosis induction by inhibiting systemic Xc- system and elevated iron levels in both cytoplasm and mitochondria [48]. Intracellular (cytoplasm and mitochondria) iron diminution by DFO or Fer-1 could inhibit ferroptosis [49]. Of interest, an obvious increase of iron levels both in cytoplasm and mitochondria was observed after exposure to both MEHP and Erastin compared to Erastin treatment (Fig. 4A-B and F-G), and these accumulations of iron were prevented after DFO treatment (Fig. 5G-H and Fig. S6A). We consistently observed that MEHP-Erastin reduced the levels of GSH-PX, T-GSH, GSH/GSSG and elevated the levels of 8-OH, LPO, MDA and 4-HNE compared to Erastin treatment (Fig. 3A-B), while these levels were alleviated after Fer-1 treatment (Fig. 5A-C). The data also indicated that MEHP could increase Erastin-induced ROS, and lipid peroxidation production in TM3 cell line (Fig. 3D-E and G-I), but Fer-1 completely prevented ROS productions (Fig. 5E-F and Fig. S6A). Moreover, the results indicated that the levels of ACSL4, PTGS2 and LPCAT3 were obviously enhanced, and the levels of FTH1, SLC3A2, SLC7A11 and GPX4 were significantly enhanced after exposure to Erastin or MEHP-Erastin groups compared to DMSO group in TM3 cells (Fig. 4C-E and Fig. S5). However, there was no obvious difference in levels of ferroptosis-related markers between MEHP-Erastin and Erastin treatment group (Fig. 4C-E and Fig. S5). Interestingly, exposure to MEHP-Erastin substantially reduced the levels of SLC7A11, GPX4 and 4-HNE, but the changes were inhibited after Fer-1 treatment (Fig. 5J-L and Fig. S6B). The mitochondrial morphology was abnormal (smaller mitochondria, vacuolated mitochondria, few mitochondrial cristae and some autophagic vesicles) in TM3 cell after exposure to Erastin. Interestingly, we assessed that the mitochondrial morphology was more abnormal with dense outer membranes, smaller mitochondria, more severe mitochondrial vacuolation, almost disappearance of mitochondrial cristae and abundant autophagic vesicles in TM3 cells after exposure to MEHP-Erastin compared to Erastin treatment (Fig. 3C and F). Nevertheless, these injuries were mitigated after Fer-1 treatment (Fig. 5D and Fig. S6A). These findings suggested that MEHP and Erastin synergized to induce ferroptosis via downregulation of SLC7A11, which disturbed glutathione system and intracellular iron levels disorders (Fig. 5I).

### SLC7A11 acts as an effector in blocking ferroptosis induced by synergistic effect of the MEHP and Eratin

**Fig. 6:**
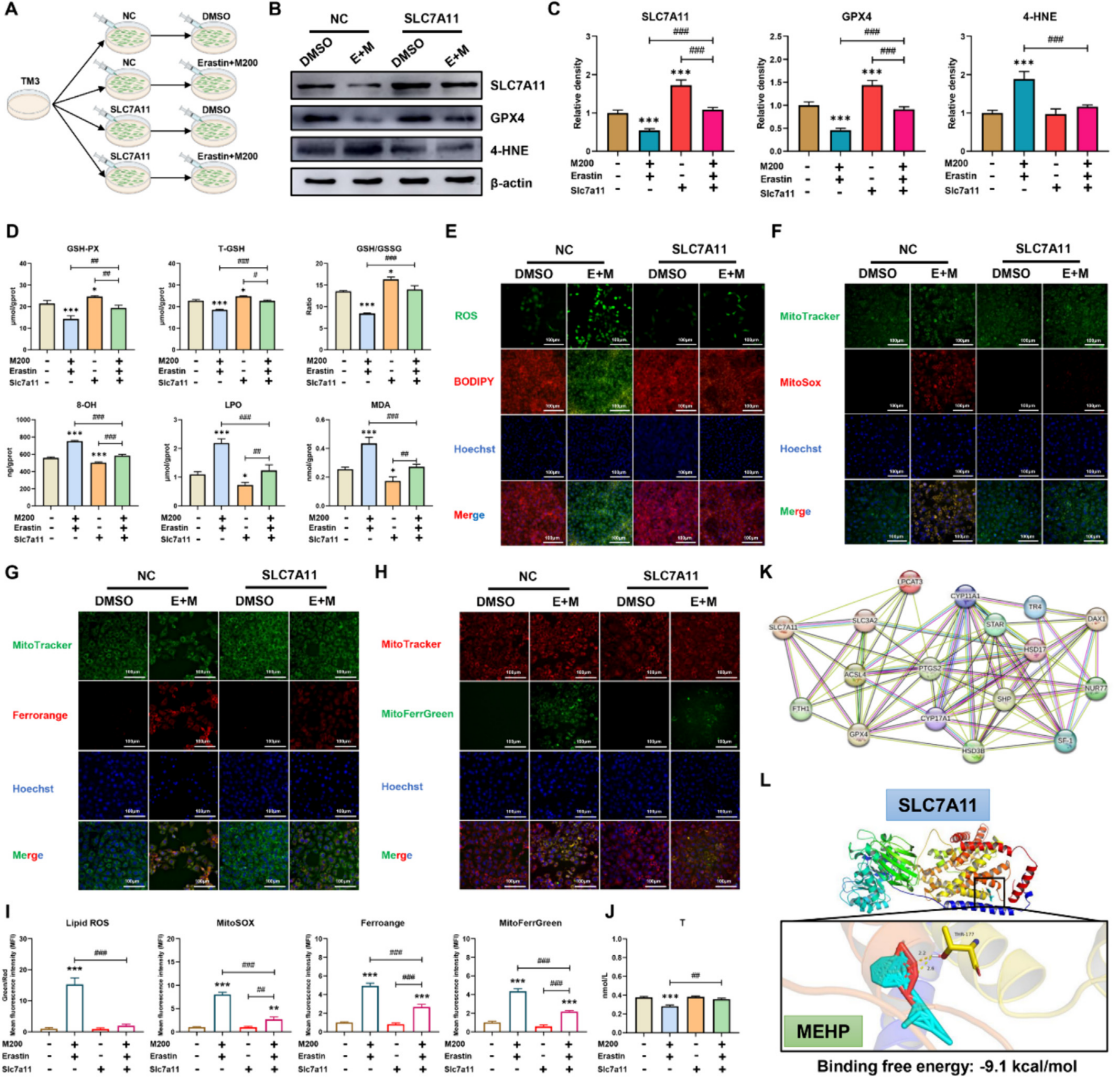
**MEHP synergistically induced ferroptosis with Erastin by suppressing SLC7A11-mediated GSH synthesis (n = 3). (A)** TM3 cells were incubated with DMSO, SLC7A11 and/or Erastin-MEHP. (B) The protein levels of SLC7A11, GPX4 and 4-HNE in TM3 cells. (C) Relative protein levels of SLC7A11, GPX4 and 4-HNE in TM3 cells. (D) The T-GSH, GSH/GSSG, GSH-PX, 8-OH, LPO, MDA levels of TM3 cells. (E) The intracellular and lipid ROS level in TM3 cells. (F) The mitochondrial ROS level in TM3 cells. (G) The intracellular iron level in TM3 cells. (H) The mitochondrial iron level in TM3 cells. (I) The MFI of Ferrorange and MitoFerrGreen in TM3 cells. (B) The T level of cellular supernatant in TM3 cells. (K) PPI network of ferroptosis and testosterone synthesis. (L) Molecular docking simulation for the ligand–protein binding of MEHP with SLC7A11. Data are presented as the mean ± SD. Symbol for the significance of differences between the DMSO group and another group: *P < 0.05, **P < 0.01, ***P < 0.001. Symbol for the significance of differences between the SLC7A11 group and SLC7A11 + MEHP + Erastin (SLC7A11 + E + M) group, or MEHP + Erastin (E + M) group and the SLC7A11 + MEHP + Erastin (SLC7A11 + E + M) group: ^##^*P* < 0.01, ^###^*P* < 0.001.

SLC7A11 is a transmembrane protein that regulates glutamate antiporter activity in Xc − system [15]. This antiporter is used to synthesize GSH by reducing cystine to cysteine, thereby maintaining the intracellular antioxidant defense system [50]. Consequently, our objective was to investigate the relationship between SLC7A11 and MEHP aggravation of Erastin-induced ferroptosis. We observed that, while MEHP-Erastin reduced GSH-PX, T-GSH, GSH/GSSG levels and increased 8-OH, LPO, MDA, GPX4, 4-HNE levels, overexpression of SLC7A11 restored these levels (Fig. 6A-D). Furthermore, MEHP-Erastin promoted ROS production, and lipid peroxidation production in TM3 cells, whereas overexpression of SLC7A11 obviously mitigated these damages. Consistently, SLC7A11 overexpression obviously restored combined exposure-induced decreased T levels and increased iron levels in both cytoplasm and mitochondria in TM3 cells (Fig. 6E-J). Therefore, overexpression of SLC7A11 alleviated the ferroptosis induced by MEHP-Erastin. The analysis of PPI network reveals that ferroptosis is correlated with testosterone synthesis (Fig. 6K). To confirm the possible interaction between MEHP and SLC7A11 at the molecular level, we carried out the protein–ligand docking analysis, which suggested a stable combination (Fig. 6L). These results revealed that SLC7A11-promoted GSH biosynthesis represents a vital mechanism in MEHP synergizing with Erastin to induce ferroptosis, leading to testosterone level decline (Graphical Abstract).

## Discussion

Phthalate is renowned endocrine disruptor that significantly contributes to the environmental and poses a potential health risk to humans, especially reproductive toxicity [51]. In recognition of the adverse impacts, DEHP is the most widely used phthalates and recognized as a priority hazardous pollutant in numerous countries [52]. In this study, we demonstrated that DEHP reduced testosterone level via inhibiting testosterone synthases and transcription factors. Several investigations have elucidated the impact of ferroptosis in male reproductive damage [53]. Here, we conducted this study a comprehensive analysis to further evaluate DEHP-induced testicular toxicity and explored the biological association between DEHP-induced Leydig cell injury and ferroptosis. We identified that MEHP and Erastin synergize to induce ferroptosis in Leydig cells by inhibiting SLC7A11-dependent pathway. Overexpression of SLC7A11 alleviated combined exposure-induced ferroptosis via modulating GSH, which enhanced the sensitivity of Leydig cells to Erastin. In this regard, this work identifies SLC7A11 as a negative regulator of synergistic effect of the MEHP and Eratin to induce ferroptosis.

The male reproduction is regulated by a coordinated interaction among hormones on the HPT axis [54]. Hypothalamus stimulates the pituitary gland to secrete gonadotropins, which are transported to the gonads to regulate steroid producing sex hormones including E_2_ and T, which regulate the reproductive process [55]. Interfering with the HPT axis by DEHP may be considered as a potential mechanism for the decrease in serum testosterone, LH and FSH levels [56]. Our study suggested that DEHP has potential to induce disorder in sex hormone levels. Testosterone biosynthesis in Leydig cells involves a variety of intermediate steroids, which are modulated by STAR, P450scc, 3β-HSD, P450C17, and 17β-HSD [57]. Inhibiting the level or activity of steroid-producing enzymes is an underlying mechanism for lowering testosterone level. In this study, we indicated that DEHP induced decline in steroid-producing genes and enzymes, resulting in testosterone biosynthesis disorder. Orphan members of the nuclear receptor (SF-1, Nur77, TR4, DAX1 and SHP) are defined as the ligand-regulated transcription factors [58]. In the current study, we indicated that DEHP inhibited orphan nuclear receptors synthesis, leading to testosterone production decrease. Furthermore, transcriptomic analyses are in accordance with the results of our data, which suggested that DEHP could induce steroid biosynthesis disorders. Collectively, the results proved that DEHP disrupts the HPT axis and orphan nuclear receptors systems, leading to testosterone biosynthesis and production disorder.

MEHP is the vital metabolite of DEHP and considered to be more toxic than DEHP. MEHP could mediate stimulation of steroidogenesis, which highlights an underlying impact of phthalates on male health [59]. MEHP induced apoptosis of Leydig cells may be related to the suppression of m6A up-regulation [60] and caused mitophagy by enhancing mitochondrial ROS to increase cytotoxicity [18]. In this study, MEHP reduced the levels of steroid-producing enzymes and orphan nuclear receptors. Leydig cells play vital roles in organisms masculinization [61]. Some prior studies suggested that DEHP caused decreased mitochondrial cristae, incomplete of mitochondrial membrane, autophagic vacuoles [62]. MEHP caused apparent mitochondrial cristae disappeared in the gonocytes, and chromatin condensation around the nucleolus [63]. Consistent with the above studies, both DEHP and MEHP could cause decreased mitochondrial cristae and incomplete mitochondrial membrane in Leydig cells. Several lines of evidence suggest that DEHP could cause structural and functional impairment in Leydig cells.

Elevated ROS is not only an important cause of ferroptosis, but also the outcome after ferroptosis [64]. Although ferroptosis is triggered by diverse agents, the pivotal characteristics of ferroptosis are mitochondrion injury [65] and lipid peroxidation [66]. To clarify the effect of MEHP in the modulation of ferroptosis, our study chose TM3 cell line as cell models. We first assessed the effect of MEHP on ferroptosis-related indicators in TM3 cells. MEHP exposure alone caused lipid peroxidation damage, but did not cause changes in ferroptosis-related proteins and iron levels both in cytoplasm and mitochondria. Interestingly, only SLC7A11 proteins was significantly reduced after exposure to MEHP. However, the combined exposure (MEHP-Erastin) was sufficient to induce ferroptosis, even more severe than exposure to Erastin alone, as evidenced by enhancement of ferrous iron and lipid peroxides, as well as exhaustion of GSH. Chelation of intracellular iron with DFO or prevention of lipid peroxidation with Fer-1 protected TM3 cell against combined exposure-induced ferroptosis. Furthermore, the results indicated that combined exposure-induced morphological changes were also consistent with the features of ferroptosis [17], whereas Fer-1 protected TM3 cell against combined exposure-induced these ferroptosis characteristics. Thus, we hypothesized that MEHP synergistically induced the occurrence of ferroptosis with Erastin.

Occurrence of ferroptosis caused by MEHP-Erastin prompted us to study its potential mechanisms. The present study revealed that MEHP-Erastin obviously inhibits glutathione system, leading to the degradation of anti-oxidative enzymes and the elevation in lipid peroxidation products. SLC7A11 is a light chain subunit that plays a vital role in modulating redox homeostasis [67]. Some researcher studies highlighted the novel role of SLC7A11 in modulating ferroptosis [68]. To further explore the molecular mechanisms through which MEHP-Erastin aggravate ferroptosis, we subsequently detected the levels of intracellular ferrous iron and lipid peroxides, as well as GSH and cysteine after SLC7A11 overexpression. Similar to the results obtained with ferroptosis inhibitors, SLC7A11 overexpression could protect TM3 cell against combined exposure-induced death, further confirming the vital role of SLC7A11-dependent pathway in this process. Furthermore, our molecular docking simulation results indicated that MEHP has strong docking ability with SLC7A11, implying that SLC7A11 may be a critical target in MEHP-induced Leydig cell injury. At present, there have been a few studies about the relationship between ferroptosis and testosterone synthesis disorder. Heavy metal cadmium exposure induced ferroptosis by iron homeostasis dysregulation, causing testicular dysfunction and testosterone synthesis disorder [69]. Glyphosate-induced cytotoxicity and testosterone synthesis inhibition are obviously prevented by NCOA4 knockdown, suggesting the key role of NCOA4-mediated ferroptosis in testosterone synthesis dysregulation [70]. In our study, the analysis of PPI network showed that ferroptosis is correlated with testosterone synthesis, and MEHP exposure reduced testosterone level in testis and Leydig cells. Therefore, our study revealed that the inhibition of SLC7A11-promoted GSH biosynthesis represents a crucial mechanism in synergistic effect of the MEHP and Erastin, which could accelerate deterioration in testosterone levels.

## Conclusion

In summary, we conclude that DEHP exposure is not sufficient to trigger ferroptosis, while the cytotoxicity is obviously increased after combined exposure Erastin in male mice, showing that MEHP synergistically induced ferroptosis with small-molecule inducers. It is reasonable that combined exposure of phthalates and other toxicants might have similar synergistic effects in the environment. Future toxicological studies understanding joint toxic impacts of toxicants will become an urgent problem. Furthermore, the present study reveals a potential protective role for SLC7A11 in ferroptosis. These findings prove that SLC7A11 may act as a probable therapeutic target for preventing ferroptosis after exposure to phthalates and other toxicants in the environment.

## Availability of data and materials

The raw data of genome and transcriptome sequencing has been uploaded into the NCBI SRA database and are accessible via the accession number PRJNA1095410.

## Compliance with Ethics requirements

All Institutional and National Guidelines for the care and use of animals were followed.

All procedures for animal experiments were performed in the Guidelines for Care and Use of Laboratory Animals of Northeast Agricultural University (NEAU). Experiments were approved by the Animal Ethics Committee (NEAUEC20220341).
