# Supplementary material for: SLC7A11 as a therapeutic target to attenuate phthalates-driven testosterone level decline in mice
Source: J Adv Res. 2024 May 24;71:369–81. doi: 10.1016/j.jare.2024.05.026 (PMC12126716; doi:10.1016/j.jare.2024.05.026)
Supplement: Supplementary Data 1 [file mmc1.docx]

***Supplemental Materials***

**SLC7A11 as a therapeutic target to attenuate phthalates-driven testosterone level decline in mice**


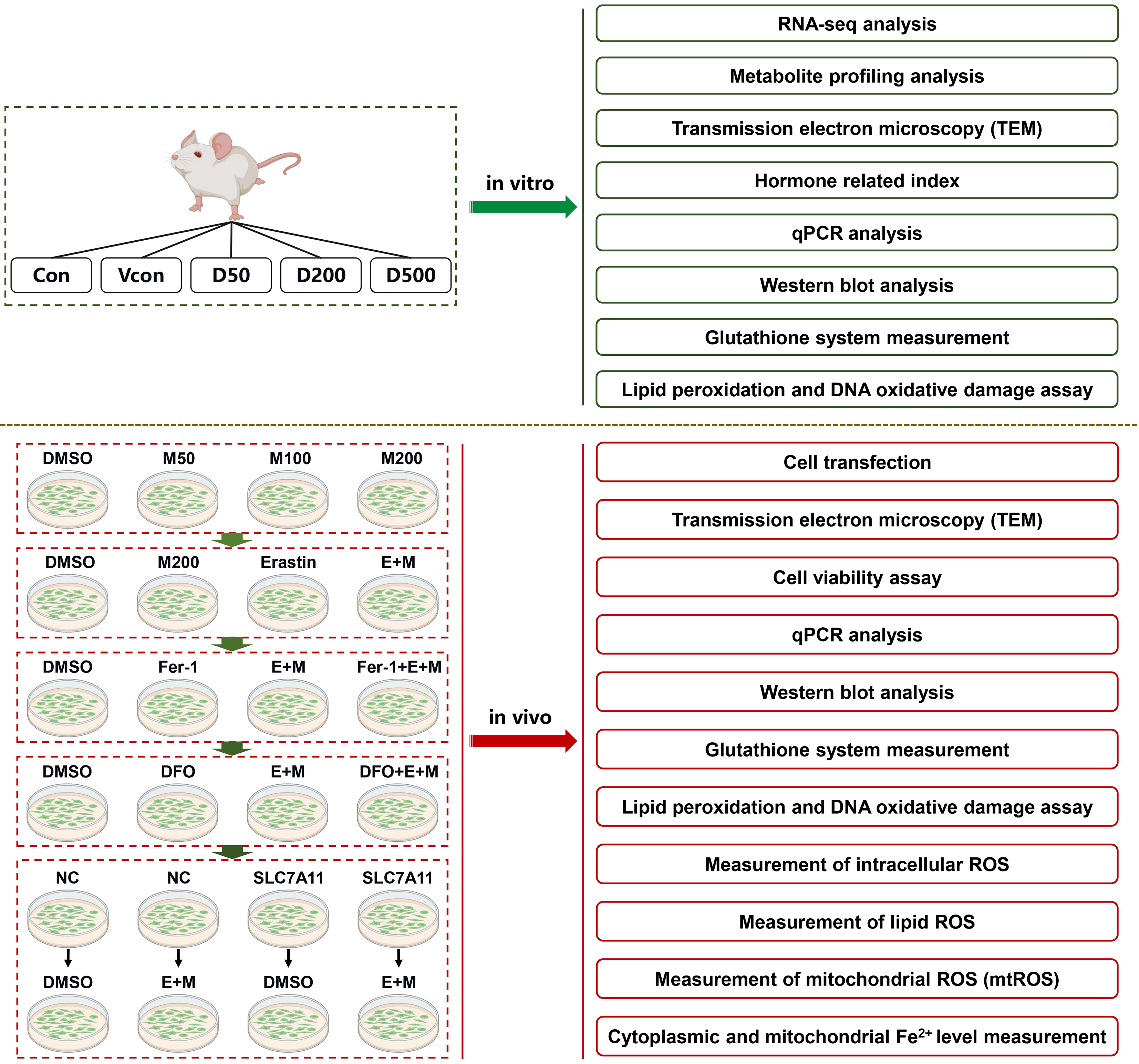


**Fig. S1. In vivo and in vitro study design.**


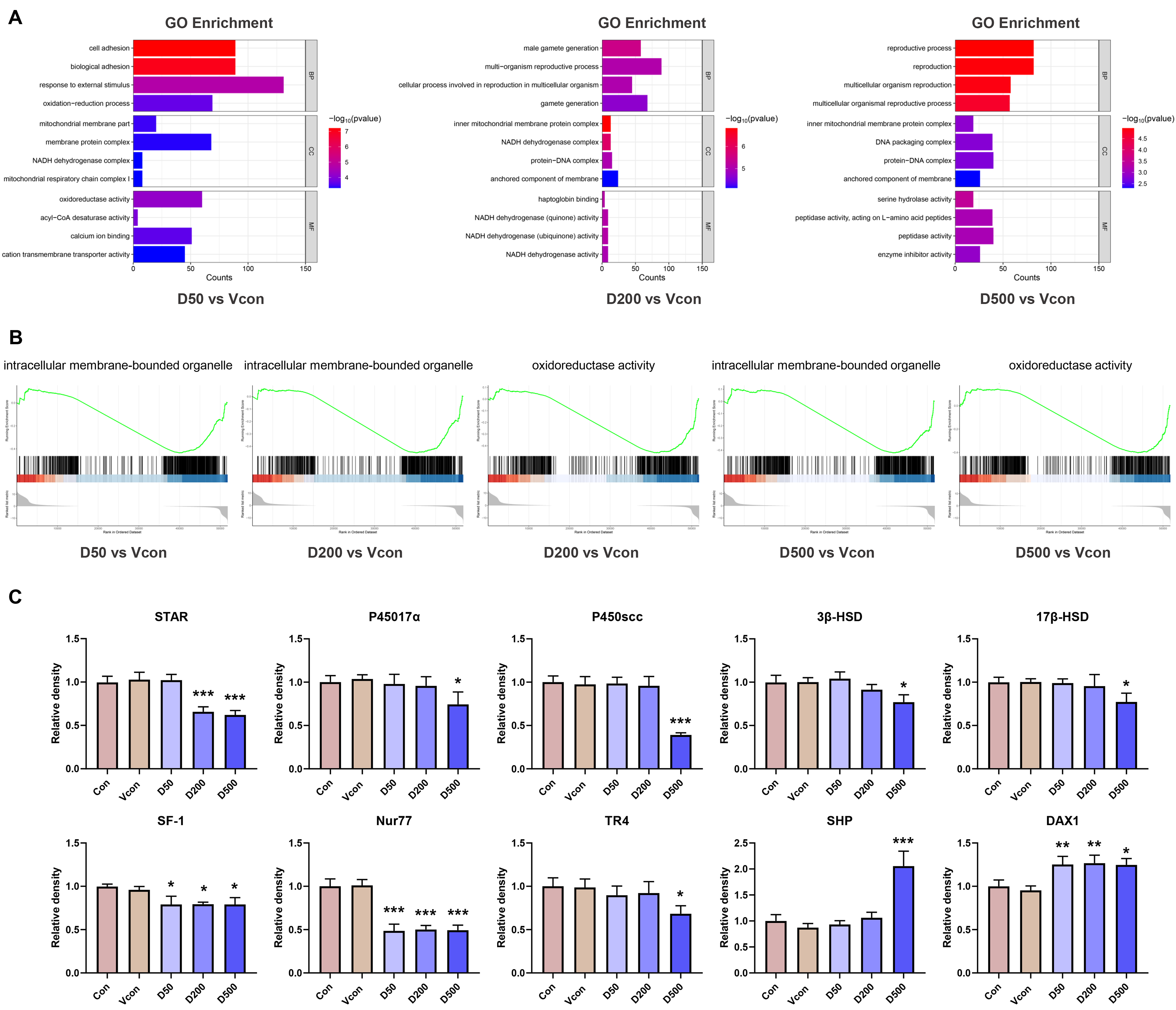


**Fig. S2. DEHP caused decreased antioxidant capacity and steroid hormone synthesis disorder.** (A) GO analysis. (B) GSEA analysis. (C) The protein levels of testosterone synthases and hormones nuclear receptors in the mice testis. Data are presented as the mean ± SD. Symbol for the significance of differences between the Vcon group and another group: **P* < 0.05, ***P* < 0.01, ****P* < 0.001.


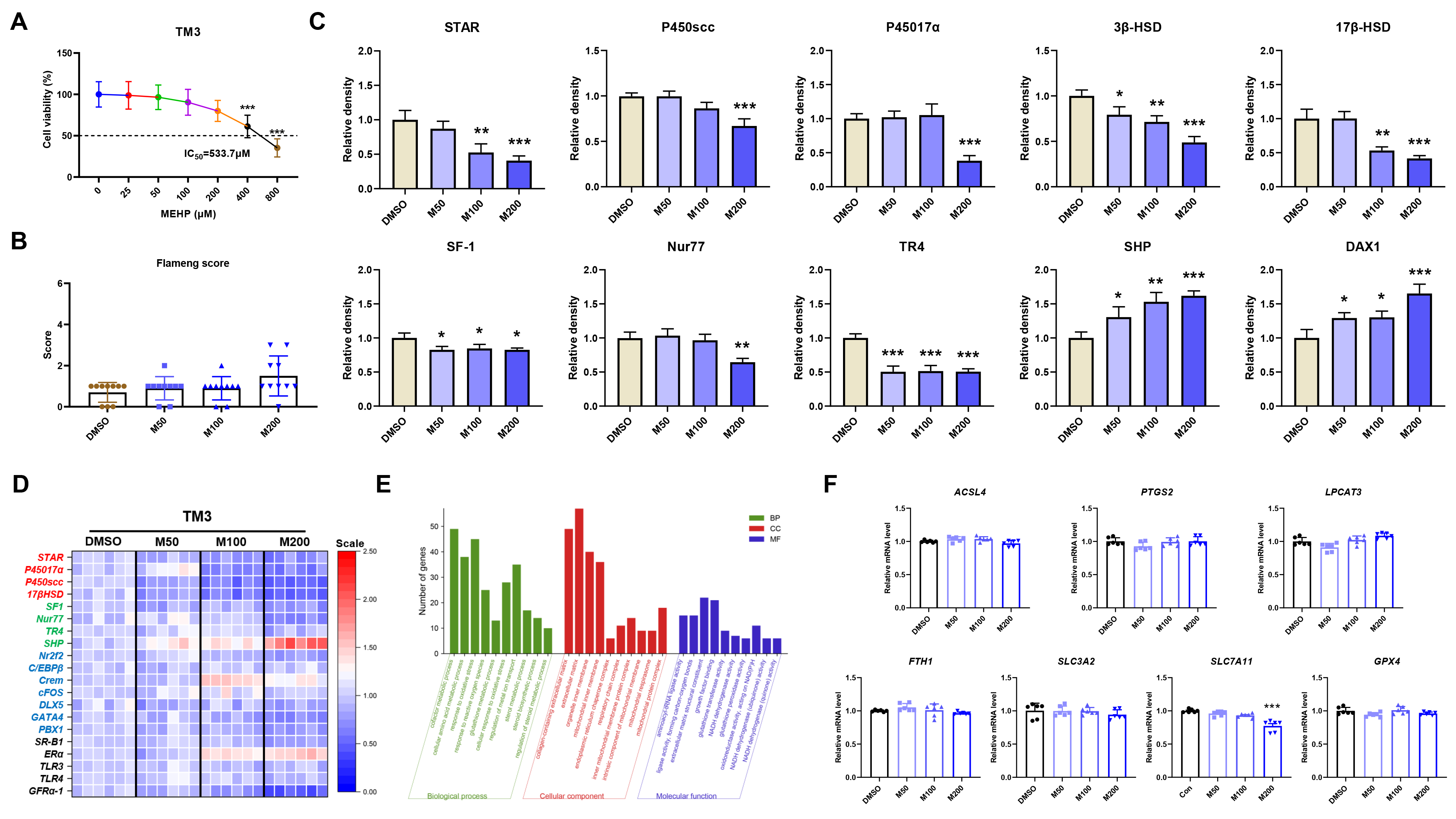


**Fig. S3. Relative mRNA and protein levels of testosterone synthesis and ferroptosis in TM3 cells after exposure to MEHP.** (A) The cell viability was assayed using CCK-8 after exposure to different concentrations of MEHP. (B) The Flameng score of TM3 cells. (C) Relative mRNA and protein levels of testosterone synthesis in TM3 cells. (D) Heatmap of relative mRNA and protein levels of testosterone synthesis in TM3 cells. (E) GO analysis. (F) Relative mRNA and protein levels of ferroptosis in TM3 cells. Data are presented as the mean ± SD. Symbol for the significance of differences between the DMSO group and another group: **P* < 0.05, ***P* < 0.01, ****P* < 0.001.


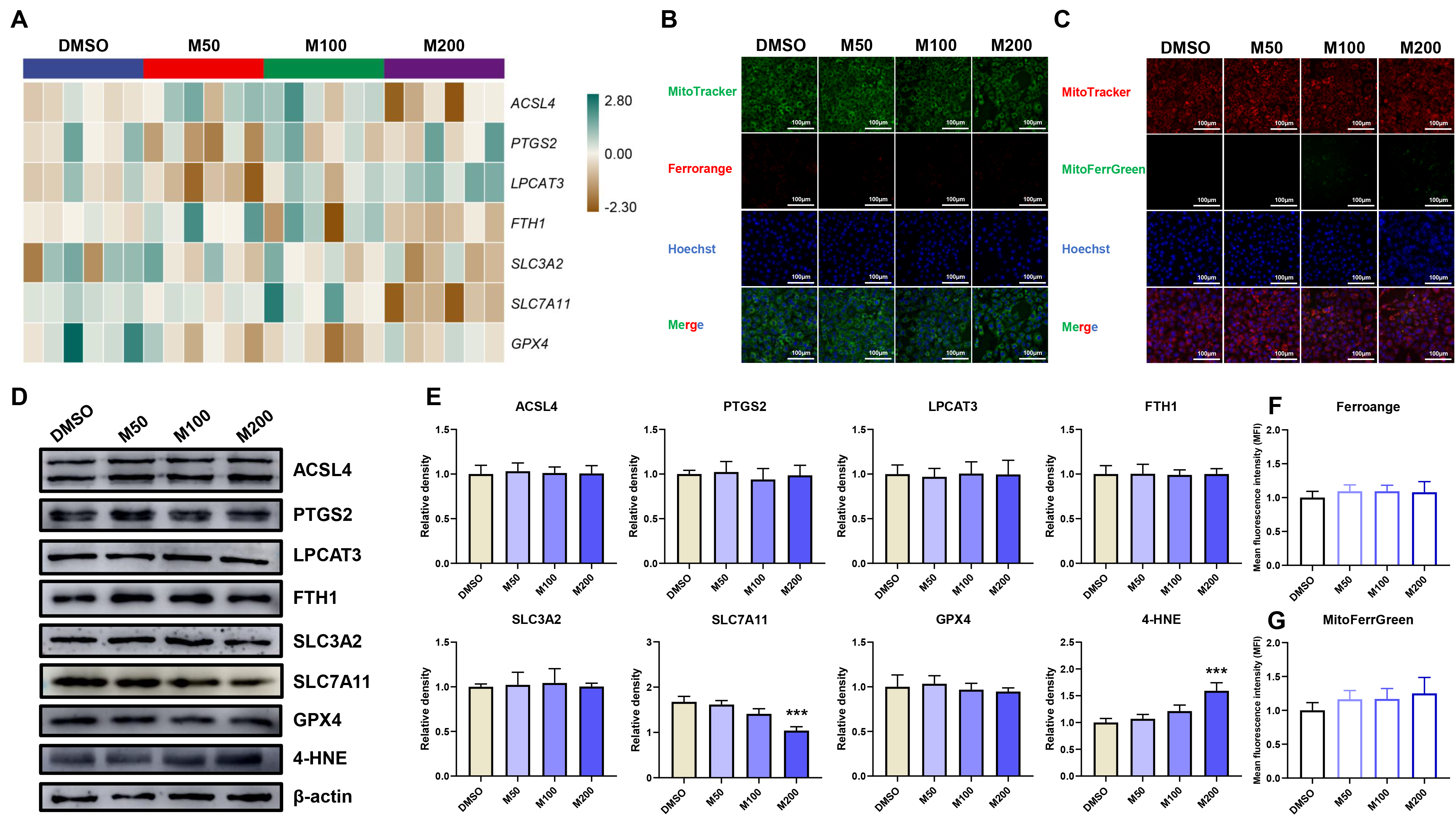


**Fig. S4. Relative protein levels of ferroptosis related indicators in TM3 cells.** (A) Heat map of relative mRNA levels of ferroptosis-related genes in the TM3 cells treated with DMSO, M50, M100 and M200. (B) The intracellular iron level in TM3 cells. (C) The mitochondrial iron level in the TM3 cells. (D) The protein levels of ferroptosis in the TM3 cells; β-actin is served as loading controls for the total fraction. (E) Relative protein levels of ACSL4, PTGS2, LPCAT3, FTH1, SLC3A2, SLC7A11, GPX4 and 4-HNE. (F) The MFI of Ferrorange. (G) The MFI of Ferrorange and MitoFerrGreen. Data are presented as the mean ± SD. Symbol for the significance of differences between the DMSO group and another group: ****P* < 0.001.


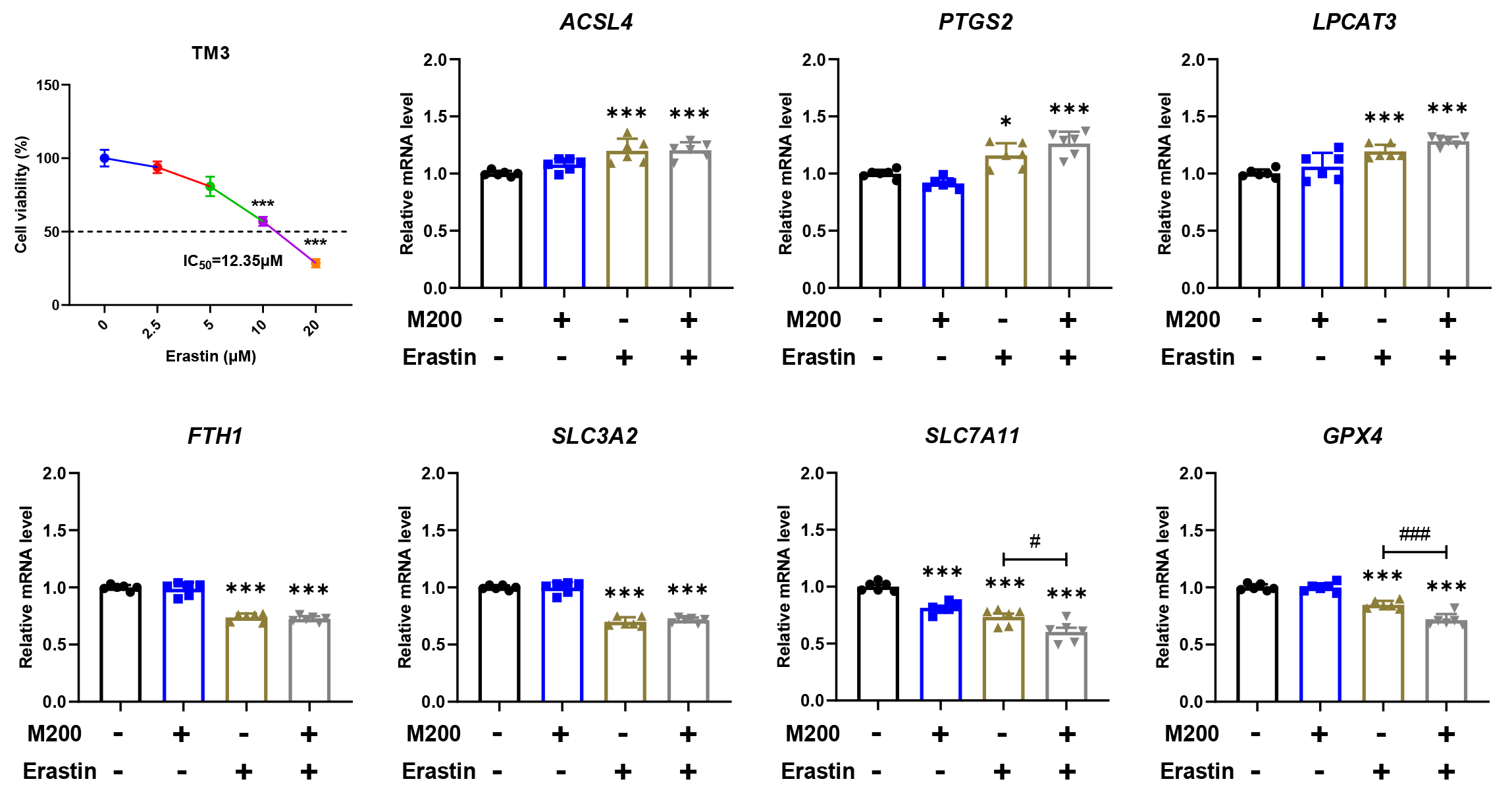


**Fig. S5. MEHP upregulated Erastin-induced ferroptosis-related proteins and iron levels in TM3 cells.** The cell viability was assayed using CCK-8 after exposure to MEHP and Erastin; relative protein levels of ACSL4, PTGS2, LPCAT3, FTH1, SLC3A2 and GPX4. Data are presented as the mean ± SD. Symbol for the significance of differences between the DMSO group and another group: **P* < 0.05, ****P* < 0.001. Symbol for the significance of differences between the Erastin group and MEHP + Erastin (E+M) group: ^#^*P* < 0.05, ^###^*P* < 0.001.

**
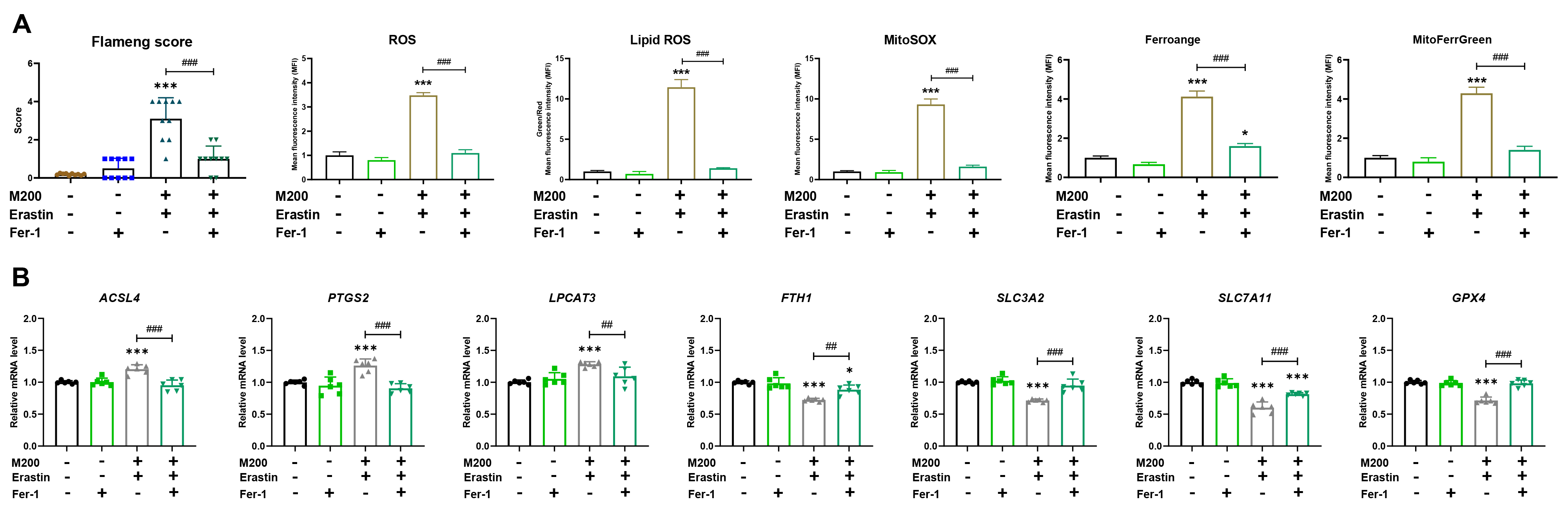
**

**Fig. S6. Effect of MEHP on oxidative stress and lipid peroxidation damage in TM3 cells.** (A) Flameng score of the TM3 cells; The MFI of ROS, Lipid ROS, MitoSOX, Ferrorange and MitoFerrGreen in TM3 cells. (B) The relative mRNA levels of ferroptosis-related genes in the TM3 cells treated with DMSO, M200 and/or Erastin. Data are presented as the mean ± SD. Symbol for the significance of differences between the DMSO group and another group: **P* < 0.05, ***P* < 0.01, ****P* < 0.001. Symbol for the significance of differences between the Erastin group and MEHP + Erastin (E+M) group: ^#^*P* < 0.05, ^##^*P* < 0.01, ^###^*P* < 0.001.

**Table S1. Animal groups*.**

| **Groups** | **Number of mice** | **Treatment** |
| --- | --- | --- |
| Group 1 (Con)^1^ | 20 | Water  Corn oil  DEHP (50 mg/kg BW^4^ /day)  DEHP (200 mg/kg BW^4^ /day)  DEHP (500 mg/kg BW^4^ /day) |
| Group 2 (Vcon)^2^ | 20 |  |
| Group 3 (D50)^3^ | 20 |  |
| Group 4 (D200)^3^ | 20 |  |
| Group 5 (D500)^3^ | 20 |  |

* Corn oil as vehicle dissolves DEHP.

^1^ Group 1 (Con): treatment control.

^2^ Group 2 (Vcon): vehicle control.

^3^ Group 3-5: DEHP treatment.

^4^ BW: Body Weight

**Table S2. Sequences of oligonucleotide primers for QRT-PCR.**

| **Gene Names** | **Sequence (5' → 3')** | **NCBI Reference Sequence** | **Amplicon size (bp)** |
| --- | --- | --- | --- |
| GADPH | GGTTGTCTCCTGCGACTTCA  TGGTCCAGGGTTTCTTACTCC | NM_001289726.1 | 183 |
| β-actin | GTGCTATGTTGCTCTAGACTTCG  ATGCCACAGGATTCCATACC | NM_007393.5 | 174 |
| LHR | CTCGCCCGACTATCTCTCAC  ACGACCTCATTAAGTCCCCTG | NM_013582.3 | 77 |
| SR-B1 | TGTACTGCCTAACATCTTGGTCC  ACTGTGCGGTTCATAAAAGCA | NM_016741.2 | 126 |
| ERα | CCTCCCGCCTTCTACAGGT  CACACGGCACAGTAGCGAG | NM_007956.5 | 128 |
| TLR3 | AAGGTACATCACGCAGTTCAGC  TCTTCGCAAACAGAGTGCAT | NM_126166.5 | 114 |
| TLR4 | GGTTTACACGTCCATCGGTT  GCAGAAACATTCGCCAAGCA | NM_021297.3 | 179 |
| SF1 | TGCCCTGTTGGATTACACCT  CCTCCACCAGGCACAATAGCAA | NM_001316687.1 | 80 |
| Nur77 | ACACTTGGACTCCGGGCCTA  TGCCCACTTTCGGATAACGTC | NM_010444.2 | 151 |
| TR4 | AGAAGTCCTCTGATAGCCACT  TGTTTCAGCCTTGGAATCCG | NM_001347342.1 | 156 |
| SHP | CAGCCATCAGACCGGCCACAA  TCAAGGCTCCAGAAAGACTCC | NM_011850.3 | 80 |
| DAX1 | GGACCGTGCTCTTTAACCC  TTCAGTGACGACATCGCTA | NM_007430.5 | 191 |
| STAR | CGGGTGGATGGGTCAAGTTC  GCACTTCGTCCCCGTTCTC | NM_011485.5 | 188 |
| P45017α | AGTCAAAGACACCTAATGCCAAG  ACGTCTGGGGAGAAACGGT | NM_007809.3 | 83 |
| P450scc | AGGTCCTTCAATGAGATCCCTT  TCCCTGTAAATGGGGCCATAC | NM_019779.4 | 137 |
| 3β-HSD | AGCTCTGGACAAAGTATTCCGA  GCCTCCAATAGGTTCTGGGT | NM_008293.4 | 234 |
| 17β-HSD | CGGGCAGACCGTTCTCATC  CCTCAACACCACGCTTATTGAT | NM_001163486.1 | 119 |
| TGFα | CAACAAGTGCCCAGATTCCC  TCACAGCGAACACCCACGTA | NM_031199.5 | 117 |
| TGF-β1 | TCCATCACTAGATCGCCCTT  ACCGACCTTTGCCAATGCTT | NM_009370.3 | 158 |
| TGF-β3 | GCCCCAAAGGAATTACCTC  TATGTGCTCATCCGGTCGAA | NM_009368.3 | 173 |
| IGF-1 | TACAAAAGCAGCCCGCTCT  TTCTCCTTTGCAGCTTCGTT | NM_010512.5 | 107 |
| Ggnbp2 | TTCAGCGACACAGTATGCTT  AGGCGTTCCACACTTCGAC | NM_001364111.1 | 127 |
| EGF | AGTTGCCCTGACTCTACCG  GCATCGATCCCCAGAATAGCC | NM_010113.4 | 186 |
| STRA8 | AAGTGTCGAAGGTGCATGGT  AGGTTGTTGAAGAGCCCTACC | NM_009292.2 | 195 |
| GFRα-1 | ACGTTTTACCACCGTGTGCT  GCGAGACCATCCTTTCCGTA | NM_010279.3 | 109 |
| Nr2f2 | AACACGGTTCGGAAAGCTCT  TTTTACCTACCAAACGGACGA | M_009697.3 | 98 |
| C/EBPβ | ACGACTTCCTCTCCGACCT  CCGAGGCTCACGTAACCGTA | NM_001287738.1 | 85 |
| Crem | CAGTCCCCAGCAACTAGCAG  TTTCAAGCACAGCCACACGA | NM_001374833.1 | 140 |
| cFOS | GCCAGTCAAGAGCATCAGCAAC  CCATCCCCAAGGAATTGCTGT | NM_010234.3 | 179 |
| Dlx6 | GCAACTCCTACAACCACCGAT  TCCCCTTTCCGTTGAACCTG | NM_010057.2 | 180 |
| Dlx5 | CTTTCAGCTGGCCGCTTT  TTTTCACCTGTGTTTGCGTCA | NM_010056.3 | 110 |
| GATA4 | CCAACTGCCAGACTACCACC  CCCTCCTTCCGCATTGCAAG | NM_001310610.1 | 127 |
| PBX1 | CTCAGTGGAGCATTCCGACT  TCCGGCTTTGCTCTCGAA | NM_183355.3 | 137 |
| PTGS2 | TGTGCGACATACTCAAGCAG  TGTTGCACGTAGTCTTCGAT | NM_011198.4 | 118 |
| FTH1 | TGCCTCCTACGTCTATCTGTC  GCTTCATCAGTTTCTCGGCAT | NM_010239.2 | 128 |
| SLC7A11 | CTATTTTACCACCATCAGTGCG  ATCGGGACTGCTAATGAGAATT | NM_011990.2 | 102 |
| GPX4 | ATAAGAACGGCTGCGTGGTGAAG  TAGAGATAGCACGGCAGGTCCTTC | NM_008162.4 | 82 |
| ACSL4 | CTCACCATTATATTGCTGCCTGT  TCTCTTTGCCATAGCGTTTTTCT | NM_207625.2 | 114 |
| LPCAT3 | GCCGTTATTACTACCCTTTGCT  ACACAGCCCAATTAGCTTCAG | NM_145130.2 | 132 |
| SLC3A2 | TGATGAATGCACCCTTGTACTTG  GCTCCCCAGTGAAAGTGGA | NM_001161413.1 | 183 |

**Table S3. Antibodies obtained from different vendors in this report.**

| **Target** | **Type** | **Vendor** | **Catalog no.** | **Usage (Dilution)** |
| --- | --- | --- | --- | --- |
| β-actin | Polyclonal | GeneTex | GTX109639  bs-3570R  A1373  A1713  A8035 | IB (1:2000) |
| STAR | Polyclonal | Bioss |  | IB (1:1000) |
| P45017α | Polyclonal | Abclonal |  | IB (1:1000) |
| P450scc | Polyclonal | Abclonal |  | IB (1:1000) |
| 3β-HSD | Polyclonal | Abclonal |  | IB (1:1000) |
| 17β-HSD | Polyclonal | Abclonal | A10839 | IB (1:1000) |
| SF-1 | Polyclonal | Abclonal | A24536 | IB (1:1000) |
| Nur77 | Polyclonal | Proteintech | 12235-1-AP | IB (1:1000) |
| TR4 | Polyclonal | Abclonal | A6422 | IB (1:1000) |
| SHP | Polyclonal | Abclonal | A16454 | IB (1:1000) |
| DAX1 | Polyclonal | Abclonal | A1740 | IB (1:1000) |
| ACSL4 | Polyclonal | Abclonal | A6826 | IB (1:1000) |
| PTGS2 | Polyclonal | Abclonal | A1253 | IB (1:1000) |
| LPCAT3 | Polyclonal | Abclonal | A17604 | IB (1:1000) |
| FTH1 | Polyclonal | Abclonal | A1144 | IB (1:1000) |
| SLC3A2 | Monoclonal | Abclonal | A3658 | IB (1:1000) |
| SLC7A11 | Polyclonal | Abclonal | A13685 | IB (1:1000) |
| GPX4 | Polyclonal | Abclonal | bs-3884R | IB (1:1000) |
| 4-HNE | Polyclonal | Abcam | ab46545 | IB (1:1000) |

Abbreviations used: IB-immunoblot analysis.

**Supplementary 1. Details of Materials and Methods**

**Supplementary 1.1. RNA-seq analysis**

Total RNA was isolated using a TRIzol total RNA extractibn kit (TIANGEN, Cat.No. DP424), which yielded > 2 μg of total RNA per sample. RNA quality was examined by 0.8% agarose gel electrophoresis and spectrophotometry. High-quality RNA with a 260/280 absorbance ratio of 1.8-2.2 was used for library construction and sequencing. Illumina HiSeq library construction was performed according to the manufacturer's instructions (Illumina,USA). Oligo-dT primers are used to transverse mRNA to obtain cDNA (APExBIO, Cat. No. K1159). Amplify cDNA for the synthesis of the second chain of cDNA. Purify cDNA products by magnetic beads. After library construction, library fragments were enriched by PCR amplification and selected according to a fragment size of 350-550 bp. The library was quality-assessed using an Agilent 2100 Bioanalyzer (Agilent, USA). The library was sequenced using the Illumina NovaSeq 6000 sequencing platform (Paired end150) to generate raw reads. Raw paired-end fastq reads were filtered by TrimGaloreto discard the adapters and low-quality bases via calling the Cutadapt tool. The clean reads obtained were then aligned to the mm10 mouse genome using HISAT2, followed by reference genome-guided transcriptome assembly and gene expression quantification using StringTie. Differentially expressed genes (DEGs) were identified by DEseq2(for sample with replications) or edgeR (for sample with no replication) with a cut-off value of log2|fold-change|>1 and p-adjust <0.05. The clusterProfiler[was used to perform functional enrichment analysis for the annotated significant KEGG pathway categories. Terms with pvalue<0.05 were considered significant.

**Supplementary 1.2. Metabolite profiling analysis**

About 50 mg of each sample was weighted out and 400μl methanol (containing 5 μg/mL 2-Chloro-L-phenylalanine as internal standard) was added to it. The mixture was mixed by vortex mixer for 1 min and homogenized for 3 min at 60 Hz for two times. Then the mixture was centrifuged at 13000 rpm, 4°C for 10 min. Supernatant was transferred to sampler vials for detected. An in-house quality control (QC) was prepared by mixing equal amount of each sample. Agilent 1290 InfinityⅡUHPLC system coupled to an Agilent 6545 UHD and Accurate-Mass Q-TOF/MS was used for LC-MS analysis. The chromatographic column used was Waters Waters XSelect HSS T3 (2.5 μm 100*2.1mm). Mobile phase: A: aqueous solution with 0.1% formic acid. B: acetonitrile solution with 0.1% formic acid. Flow rate: 0.4 ml/min. Column temperature: 40°C. Injection volume: 4 μl. Gradient elution condition optimized: 0-3min, 20% B;3-9min, 20-95% B;9-13min, 95% B;13-13.1min, 95-5% B;13.1-16min, 5% B. Raw data were converted the common (mz.data) format by Agilent Masshunter Qualitative Analysis B.08.00 software (Agilent Technologies, USA). In the R software platform, the XCMS program was used in peak identification, retention time correction, automatic integration pretreatment. Then the data were subjected to internal standard normalization. Visualization matrices containing sample name, m/z-RT pair and peak area was obtained. 2045 features were got in positive mode and 2764 features in negative mode. After qualitative analysis, the data matrices were import into R and multivariate analysis was conducted.

**Supplementary 1.3. Flameng score criteria**

The mitochondrial Flameng score criteria under transmission electron microscope is as follows. The greater the myocardial mitochondria damage, the higher the score and injury is scored as 0–4 points: 0, the structure of mitochondria is normal and they are full of particles; 1, the structure of mitochondria is essentially normal, but the matrix particles are lost; 2, mitochondrial swelling and matrix transparency are apparent; 3, rupture of mitochondrial cristae with matrix transparency and concentration; 4, the mitochondrial cristae are split, the integrity of the mitochondria inside and outside the membrane has been lost, and they appear vacuolated.

**Supplementary 1.4. qRT–PCR**

2.1 Isolate RNA

2.1.1 Homogenization

The tissue and cell samples in 1 mL of TRIzol per 50-100 mg of tissue using homogenizer.

2.1.2 Phase Separation

Add 0.2 mL of Chloroform substitutes per 1 mL of RNA Extraction. Cap sample tubes securely. Vortex samples vigorously for 15 seconds and incubate them at room temperature for 2 to 3 minutes. Centrifuge the samples at 12,000 rpm for 15 minutes at 4°C. Following centrifugation, the mixture separates into lower phenol-chloroform phase, an interphase, and a colorless upper aqueous phase. RNA remains exclusively in the aqueous phase. Transfer upper aqueous phase carefully without disturbing the interphase into fresh tube.

2.1.3 RNA Precipitation

Precipitate the RNA from the aqueous phase by mixing with isopropyl alcohol. Incubate samples at -20℃ for 15 minutes and centrifuge at 12,000 rpm for 1 minutes at 4℃.

2.1.4 RNA Wash

Remove the supernatant completely. Wash the RNA pellet once with 75% ethanol. Mix the samples by vortexing and centrifuge at 12,000 rpm for 1 minutes at 4°C. Remove all leftover ethanol.

2.1.5 Redissolving RNA

Air-dry RNA pellet for 3-5 minutes. It is important not to let the RNA pellet dry completely as this will greatly decrease its solubility. Then add 15μL RNA Storage Solution.

2.1.6 Spectrophotometric Analysis

Take OD at 260 nm and 280 nm to determine sample concentration and purity. The A260/A280 ratio should be between 1.8 and 2.1.

2.2 First Strand cDNA Synthesis

2.2.1 Add the following reagents (TransGen Biotech) into a sterile, nuclease-free tube on ice in the indicated order: Template RNA 1 µg, 5×SweScript All-in-One SuperMix for qPCR 4 µL, gDNA Remover1 µL, nuclease-freeWater to 20 µL, total volume 20 µL.

2.2.3 Mix gently and centrifuge briefly.

2.2.4 Incubate for 45 min at 42°C.

2.2.5 Terminate the reaction by heating at 85°C for 5 seconds.

**Supplementary 1.5. Western blot**

The testicular tissues and TM3 cells were lysed in RIPA buffer (Beyotime, China) with protease inhibitor cocktail (MedChem Express, USA). After protein quantification, the proteins of testicular tissue were loaded and resolved by SDS-PAGE electrophoresis and transferred to nitrocellulose filter membrane, which were then blocked with PBST/Milk 5% for 2 h and incubated at 4 °C with primary antibodies (Abcam, England; CST, USA; ABclonal Technology, China; Biosynthesis Biotechnology Co., Ltd., China) overnight. These Membranes were incubated with secondary antibody (Zhongshan Jinqiao Biotechnology Co., Ltd., Beijing, China) for 50 min at 37 °C. The proteins were visualized by using Amersham Imager 600 (GE, Switzerland). The protein densitometry was performed by using Image J software.

**Supplementary 1.6. Molecular docking**

PubChem and PDB were used to find the chemical and conformational information of the relevant proteins (SLC7A11) and small-molecule compounds (MEHP). The AutoTools software was used to remove the redundant protein chains, ligands and water molecules with hydrogenation before running docking experiments. The AutoGrid software was used to calculate the energy lattice points. AutoDock Vina was used to simulate the docking condition between proteins (SLC7A11) and small molecules (MEHP). Autogrid calculations were performed, selecting AutoDock Vina as the docking algorithm, and AutoDock was used for molecular docking. A binding energy of less than −9.1 kcal/mol was selected as the reference, and the binding energy results were visualized using PyMOL.
